# Community participation for reproductive, maternal, newborn and child health: insights from the design and implementation of the BornFyne-prenatal management system digital platform in Cameroon

**DOI:** 10.3389/fdgth.2023.1218641

**Published:** 2023-08-17

**Authors:** Pamela Obegu, Miriam Nkangu, Ngo Valery Ngo, Franck Wanda, Mwenya Kasonde, Odette D. Kibu, Nelly Abong, Victor Ndiforchu, Arone Wondwossen Fantaye, Amos Buh, Ronald M. Gobina, Denis A. Foretia, Nkengafack Fobellah, Sanni Yaya

**Affiliations:** ^1^ Health Promotion Alliance of Cameroon, Yaounde, Cameroon; ^2^ School of Epidemiology and Public Health, University of Ottawa, Ottawa, ON, Canada; ^3^ Nkafu Policy Institute, Denis and Lenora Foretia Foundation, Yaounde, Cameroon; ^4^ Faculty of Medicine, University of Ottawa, Ottawa, ON, Canada; ^5^ Interdisciplinary School of Health Sciences, University of Ottawa, Ottawa, ON, Canada; ^6^ Center for Multicultural and Global Health, University of Tennessee Health Science Center, Memphis, TN, United States; ^7^ School of International Development and Global Studies, University of Ottawa, Ottawa, ON, Canada

**Keywords:** digital health, stakeholders, RMNCH, low-middle income countries, Cameroon

## Abstract

**Introduction:**

Across communities in low-middle income countries, digital health is currently revolutionizing the delivery of health services, particularly in the field of reproductive, maternal, newborn, and child health (RMNCH) services. While studies have shown the effectiveness of mHealth in delivering RMNCH services, there is little information about factors that enhance mHealth services utilization in low-cost settings including stakeholders’ level of influence on the implementation of digital health intervention in sub–Saharan Africa. This paper seeks to describe important lessons on the levels of stakeholders’ direct or indirect influence on the design and implementation of the BornFyne-PNMS digital health platform to support RMNCH services.

**Methods:**

A participatory research (PR) design approach was employed to explore stakeholders’ perspectives of a new initiative, through direct engagement of local priorities and perspectives. The process of introducing the digital application called the BornFyne-PNMS for district health delivery system and the community, and integrating it within the district health delivery system was guided by research-to-action, consistent with the PR approach. To explore stakeholders’ perspectives through a PR approach, we conducted a series of stakeholder meetings fashioned after focus group discussions.

**Results:**

Issues around male involvement in the program, sensitization and equity concerns arose. Emergent challenges and proposed strategies for implementation from diverse stakeholders evidently enriched the design and implementation process of the project intervention. Stakeholder meetings informed the addition of variables on the mobile application that were otherwise initially omitted, which will further enhance the RMNCH electronic data collection for health information systems strengthening in Cameroon.

**Discussion:**

This study charts a direction that is critical in digital health delivery of RMNCH in a rural and low-income community and describes the important iterative stakeholder input throughout the study. The strategy of stakeholders’ involvement in the BornFyne PNMS implementation charts a direction for ownership and sustainability in the strengthening of Cameroon's health information system.

## Introduction

Available evidence has highlighted that the sustainability of community health interventions is largely dependent on the participation of stakeholders (1, 2), through the roles they play when their needs are first understood. In other words, for community-based health interventions to work, there is the need to understand the interests, attitudes, and contestations of key stakeholders (2, 3), especially in low-middle income countries (LMICs). Across communities in LMICs, digital health is currently revolutionizing the delivery of health services, particularly in the area of reproductive, maternal, newborn, and child health (RMNCH) services (4, 5). The use of mobile health (mHealth) intervention—a subcategory of digital health, has seen an increase in service utilization for RMNCH services delivery (4, 6, 7). In developing countries, this is greatly facilitated by the high penetrance of mobile phone subscription where 90% of all adults own or have access to a mobile phone subscription (6, 7). mHealth interventions range between Short Messaging Service (SMS) reminders to guide clients with information and help seek medical care from the comfort of their homes, and rapid emergency response in the event of an emergency (7, 8). These mHealth interventions reduce the myriads of constraints such as distance, time, access that are typical of RMNCH service delivery in sub-Saharan Africa (8, 9). While studies have shown the effectiveness of mHealth in delivering RMNCH services (4, 7–9), there is little information about stakeholder's involvement in the design and implementation of mhealth interventions including factors that enhance mHealth services utilization in low-cost settings, as access to mobile phones does not guarantee RMNCH service utilization (1, 10).

In recent years, health systems and interventions have been improved by the involvement of stakeholders who necessitate the development and implementation of guidelines (11). Commonly described as any “individual or group who is responsible for or affected by health and healthcare-related decisions that can be informed by research evidence,” (11, 12) stakeholders fit the “target groups” required for formative research and usability testing, as outlined in mHealth reporting guidelines (13).

In 2018, BornFyne-Prenatal Management System (PNMS) was tested as a proof-of-concept in Bali health district in Cameroon (10). BornFyne prenatal management system (PNMS) is an innovative digital health solution that targets two distinct users—pregnant women and households defined as user1 (U1), health care providers and health facilities defined as user 2 (U2). The BornFyne-PNMS platform includes: (1) A user-facing smartphone application with 6 modules (2 offline and 4 online) using a simple graphic interface. The client-facing smartphone application designed with a graphical interface for easy navigation by women with low literacy. The 2 offline modules allow users to engage with educational health content on family planning, malaria, COVID-19, antenatal and postnatal care (ANC/PNC), and any other locally relevant health information. The two 2 offline modules allow the user (U1) to listen to pre-recorded audio content in the local dialect on family planning and other priority health topics. This serves as a continuous educational platform. The content is developed following extensive user engagement to identify priority topics and user concerns and is available in various local languages. These messages are audio recorded and uploaded in addition to supplemental links for those who can read in English and French. The 4 online modules allow the user to receive personalized ANC and PNC reminder messages (according to their unique medical profile) and to contact a health worker for triage and linkage to emergency transport services in the case of an emergency (U1). (2) A health worker-facing electronic medical record that structures data entry during antenatal and postnatal visits for more complete, accurate and timely data collection. This has been designed in accordance with the WHO adaptation kit for antenatal care guidelines to support health workers in their adherence to protocols and clinical decision-making (U2) (14). In 2022, the BornFyne-PNMS project was expanded to incorporate the French districts as the project integrates components of the WHO digital adaptation kit (DAK) for antenatal care into the platform (14). The project was locally branded by the community during the proof of concept (10) as BornFyne. BornFyne in the most widely spoken pidgin English language in Cameroon and west Africa means, “safe delivery”. It could also be interpreted in many other ways, including ‘giving birth is a good thing’, and/or ‘bringing life on earth is good’.

In the context of the BornFyne-PNMS intervention (10), we defined stakeholders as organizations and individuals that are involved in the conceptualization, design, development, adoption, implementation and monitoring of the RMNCH community digital health project by way of providing, consuming, managing and/or evaluating the project activity. Stakeholders play a vital role in introducing and/or implementing a new intervention by engaging them to understand the role and perspectives towards an intervention (1). Reconciliation of their interests informs relevant modification, and refinement of intervention implementation, that enhances systemization and ownership (11, 15, 16). By identifying relevant stakeholders, a measure of the degree of their interests must be ascertained, as that goes to inform implementation upwardly or otherwise (17); hence the essence of stakeholders’ engagement—the crux of priority setting (15–17).

Following the WHO call to action for digital health (18), countries in sub-Saharan Africa are launching their digital health frameworks to align with the WHO 2018 digital health resolution agreed upon by member states as part of the strategy towards achieving Universal Health Coverage (19). There is limited research in the literature on stakeholders’ level of influence on the implementation of digital health intervention in sub-Saharan Africa (10). This paper seeks to describe important lessons on the levels of stakeholders’ direct or indirect influence on the design and implementation of the BornFyne-PNMS digital health platform to support RMNCH services. The involvement of stakeholders in digital health interventions can be complex by way of their different views, interest, and values (11, 15). Our aim of stakeholders' meetings as a first step towards introducing the BornFyne-PNMS digital health intervention and later during the implementation was to elucidate ideas from both potential consumers of the intended digital intervention and key community leaders. These stakeholders will be based in the district health delivery level and central level. Their perspectives of the new intervention to be implemented in their communities and districts have great potential in enhancing solidarity and commitment towards implementation, uptake, and sustainability.

## Methods

### Study setting

Cameroon is a lower-middle income country in Central and West Africa. It has a decentralized health system with a pyramidal structure comprising of the operational, regional, and central levels (20). The health policies are defined at the central level by the Ministry of Public Health. The regional level translates the health policies into operations, and the district level operationalizes the health policies into action (20). Cameroon is a bilingual country made up of French and English regions. There are 10 regions, 8 regions are predominantly French and 2 are predominantly English. The project is implemented in four districts in Cameroon, two from French and two from the English regions, namely, Bangem and Tiko (English, Southwest region) and Akonolinga and Ayos (French, Central region). These districts were purposefully selected to ensure they represent the two distinct languages (French and English) and a mix of rural and urban settings as defined in the context.

### Study design

As part of a larger study, this study employed a participatory research (PR) design approach to explore stakeholders’ perspectives of a new initiative, through direct engagement of local priorities and perspectives (21). The process of introducing the digital application called the BornFyne-PNMS for district health delivery system and the community with the goal of integrating it within the district health delivery system was guided by research-to-action, consistent with the PR approach (21). To explore stakeholders’ perspectives through a PR approach, we conducted a series of stakeholder meetings fashioned after focus group discussions (22).

### Ethics

Ethical approval for this study has been obtained from the National Ethics Board of Cameroon Ref 2022/07/1467/CE/CNERSH/SP and the University of Ottawa Social Science Ethics Board Ref # H-05-22-8077. Administrative clearance was received from the Ministry of Public Health at the national level in Cameroon D30-1440 No. 631-3822, in collaboration with the Division for Health Operations Research (DROS) in Cameroon, the Southwest Regional Delegation of Public Health ref # P412/MINSANTE/SWR/RDPH/CB:PF/941/618, and Central Regional Delegation of Public Health were also received ref# 1393-4/AAR/MINSANTE/SG/DRSPC. Lastly, there was a memorandum of understanding with all the district medical officers in the respective districts. Participants were voluntarily enrolled in the study on the basis of a free and informed consent. Participants were informed that information collected from the research project would be used to inform the development of a mobile application that aims to improve their use of digital health for RMNCH service delivery for better outcomes in their community.

### Study framework

We employed Hyder et al. (23) framework for our stakeholder analysis. This framework outlines considerations that generate findings from stakeholders to inform initial strategies applicable in planning, implementing, and analyzing the phases of health interventions in LMICs. In 2013, Namazzi et al. (24) adapted and modified the above stakeholder analysis approach for a maternal and newborn health (MNH) project in Uganda—an LMIC with similar sociocultural settings as Cameroon. Adapting Namazzi and colleagues’ stakeholder analysis 3-phased framework (24) for our paper, we used this framework to identify and describe the influence of stakeholders in implementing our BornFyne PNMS digital health intervention. The 3-phased stakeholder analysis framework is described as follows: (1) an identification and categorization of stakeholders, and (2) summarily mapping out stakeholders’ influence and acceptability of the BornFyne PNMS project intervention in an analytical grid (see Table 1). It also involved articulating the current level of power/influence and current level of support for each identified stakeholder on a 3-point scale (high-level influence; moderate level influence; low-level influence; and high-level supporter; moderate level supporter; low-level supporter), using terms such as: opinion leader; decision maker (24). However, we modified the third phase to incorporate both frameworks in identifying the main concerns of each stakeholder about the intervention as originally outlined by Hyder et al., and the development of an appropriate strategy on how best to interact and engage these stakeholders, as Namazzi and colleagues did.

**Table 1:** Summary of stakeholder's characteristics, influence, and acceptability of the BornFyne PNMS project intervention.

	Interest in the project	Current level of power/influence	Type of power relationship with the project setup	Current level of agreement/acceptability of proposed project intervention	Classification of stakeholder support level	Proposed strategy to deal with the stakeholder
Women	Co-design, and connected to a health facility and provider	High level influence	Beneficiaries	Strong agreement	High level supporter	Involved in project and empowered
	Receive reminder messages for ANC.	High level influence	Beneficiaries	Strong agreement	High level supporter	Involved in project and empowered
	Continuous educational platform (family planning).	High level influence	Beneficiaries	Strong agreement	High level supporter	Involved in project and empowered
	Reduce commuting time and save cost.	High level influence	Beneficiaries	Strong agreement	High level supporter	Involved in project and empowered
	Monitor for safe delivery for pregnant women at minimum cost and facilitate access	High level influence	Beneficiaries	Strong agreement	High level supporter	Involved in project and empowered
	Facilitate emergency	High level influence	Beneficiaries	Strong agreement	High level supporter	Involved in project and empowered
Men	Co-design as husbands/partners of pregnant women have a say on where their wives go for ANC visits & family planning	High level influence	Beneficiaries	Strong agreement	High level supporter	Involved in project and empowered
Health care providers (including CHW)	Co-design & implementation as health workers within the facility	Moderate level influence	Beneficiaries	Strong agreement	High level supporter	Involved in project
Central Stakeholders	Ministry of public health and other stakeholders engaged in RMNCH in Cameroon and influence policy	High level influence	Decision maker	Strong agreement	High level supporter	Continuous engagement
Regional stakeholders	Regional delegations of ministry of health	High level influence	Decision maker	Strong agreement	High level supporter	Continuous engagement
District health team	Co-design and co-implement the project	Moderate level influence	Beneficiaries	Strong agreement	High level supporter	Involved in project
	Supervise health providers, generate data, and inform policy					
Community leaders (chiefs and mayor)	Regarded as the voice of the people and convener of community members	High influence	Decision maker	Strong agreement	High level supporter	Continuous engagement
Church leaders	Faith-based organizations have strong relations with women who are often faith ‘practitioners’	High level	Opinion leaders	Moderate agreement	Moderate level supporter	Continuous support
Communication focal persons	Representatives of some non-for-profit organizations with interest in community development	Low influence	Opinion leaders	Strong agreement	Moderate level supporter	Involved in project
Research team	Co-design & Implementation of project	High influence	Mediator	Strong agreement	High level supporter	Continuous support

An analytical grid highlighting identified stakeholders’ interests and acceptability of BornFyne-PNMS intervention [Adapted from Namazzi et al. (24)].

### Identification of stakeholders

The study involved a number of stakeholders identified and categorized based on common interests toward the intervention and they are as follows: (1) primary providers—those who are involved in the co-design, planned the intervention and monitored its implementation including the BornFyne team, (2) secondary providers—those who are involved in administering the intervention and interfacing with targeted beneficiaries, (3) primary recipients—those involved in co-design and are represented as targeted beneficiaries with the “potential to benefit, weaken or strengthen the intervention” (23, 24), and (4) secondary recipients—those who are not direct beneficiaries of the intervention but are trained to interact with the beneficiaries through the 2-way interactive mobile application and to collect RMNCH data through the mobile application; and others who were somewhat involved by way of their proximity with the selected communities. Stakeholders are identified at three levels in line with the health system structure.

### District level

The BornFyne-PNMS team presented an overview of the study protocol and expectations to the district team. The district team discussed the roles and rationale for each potential stakeholder to be invited with the BornFyne PNMS team. Letters of invitation were sent to the identified stakeholders under the leadership of the district medical officers from the participating districts accordingly. Word-of-mouth invitations by community health workers went out to help identify pregnant women to attend the meetings as it concerned their health. Attendees consented to be part of the meeting when contacted and attendance was registered.

### Regional level

Following the submission of administrative approval and presentation of the project protocol and overall goals and objectives to the regional delegation of health of the two regions, the team at the delegations and the districts assisted in identifying relevant stakeholder for the meeting. Given that most of the stakeholders identified at the region could be represented at the district, we merged regional stakeholders with the district team.

### Central level

For the central-level stakeholder meeting, the BornFyne PNMS team contacted the Department of Family Health at the Ministry of Public Health in Cameroon and shared a copy of the project protocol alongside ethics approvals. The said Department reviewed the protocol, and, in their response, they indicated their interest in supporting the project's effort towards addressing maternal mortality and strengthening the health management information system. The Department then partnered with the BornFyne-PNMS to facilitate project implementation. Thus, the Department of Family Health listed relevant stakeholders representing different ministries and/or departments directly or indirectly responsible for RMNCH to be engaged with on the project. Official letters of invitation were sent out to the stakeholders under the Leadership of the Ministry of Public Health (in partnership with the BornFyne PNMS team).

### International level

Additionally, informal meetings were held with other international stakeholders, including the focal persons for RMNCH at the local WHO office in Cameroon and the WHO Digital Unit in Geneva, with the primary objective to inform and engage them on the objective of the BornFyne PNMS as the team set out to integrate the components of the WHO digital adaptation kit within the BornFyne PNMS platform. Thus, the WHO country office in collaboration with the Department of Family Health in Cameroon, invited the BornFyne-PNMS team to a national monthly coordination meeting to present the BornFyne-PNMS project and its objectives.

### Data collection

Stakeholder meetings were conducted from July to September 2022. Project goals, objectives, planned interventions, and progress of implementation were presented to stakeholders. MN, VN, NV, NF, OK, FW co-led the stakeholder discussions at the districts, regional, and central levels. MN and MK coordinated the discussions at the international level. In the fashion of focus group discussions, stakeholders were engaged to discuss their perspectives, potential challenges, and facilitators towards the planned intervention and implementation (21, 22). Informed consents were collected verbally from the stakeholders. Discussions lasted between 1.5–2 h long and were audio-recorded. Notetaking and body language (including postures and gestures of stakeholders) were observed during discussions to enrich the verbal communication/data collected (25). Discussions were held with all four categories of stakeholders (primary providers, secondary providers, primary recipients, and secondary recipients) from the four health districts. These health districts are made up of health areas and key representatives of each health area were invited. Both public and private health facility representatives were present for each district including the district medical officers, health office manager, doctors, nurses and midwives, community health workers, men, pregnant women, and breast-feeding mothers, etc. (see Table 2 below). In addition to the aforementioned stakeholders, one-on-one informal meetings were held with divisional officers from the regions and the Ministry of Public Health, Cameroon.

**Table 2:** Stakeholders identified and engaged for the BornFyne project Implementation.

Category	Stakeholder designation	Number of participants
Primary providers	Project Team (Research Investigators and project team)	11
Secondary providers	Health Providers & District Health Team (Medical Doctors, District Medical Officers, Nurses, Midwives, and Community Health Workers)	58
Primary recipients	Women (Pregnant Women and Breastfeeding Mothers) & Men (Husbands/Partners of Women of Focus)	12
Secondary recipients	Health Providers (Medical Doctors, Nurses, and Midwives)	30
	Mayor, Chiefs, Church Leaders, Divisional Officers, and Representatives from Independent Supporting Organizations (such as WHO and another International Organization), directorates, administrators	5

### Data analysis

The meetings were audio-recorded, and recordings were translated and transcribed verbatim into English language (UK). By the tenets of qualitative data analysis (22), all textual data were analyzed in three steps: (1) an initial in-vivo line-by-line coding was carried out on all stakeholders’ transcripts using MAXQDA 2020 software version, by two independent coders (MN, PO). Codes were collapsed into categories. Agreed codes were then collapsed into themes that were later interpreted into relevant concepts as reported in the results section. (2) an analytical grid adapted from Namazzi and colleagues was developed, mapping out identified influence, support and acceptability of the BornFyne PNMS project intervention (24, 26). This helped us to further understand the relationship of interests and influence within the intervention and its interaction with the health system in Cameroon. Additionally, emergent challenges and proposed solutions from the stakeholders were also highlighted. (3) Thematic analysis from the collapsed codes were further guided by the tenets of qualitative thematic analysis guide (22) to elicit the perspectives of stakeholders to better inform the implementation of the intervention drawing from the generated themes. Although we used a framework that highlighted the key areas of our study objective which was to describe important lessons on the levels of stakeholders’ direct or indirect influence on the BornFyne-PNMS intervention in the area of RMNCH in an LMIC, we also looked out for new themes emerging outside this framework (24). The themes identified were in line with the key issues that our paper sought to address, such as support for the intervention, feedback into the design and implementation challenges and solutions/strategies highlighted by stakeholders, and involvement/participation in the project activity.

We articulated the current level of agreement/acceptability of the proposed project on a 3-point scale as described by Hyder et al.: strong agreement—characterized by unwavering agreement during the discussed project plan and involvement in proposed intervention; moderate agreement—characterized by a little hesitation in body language or verbal communication concerning the project intervention; and low agreement—indifference or outright disagreement toward involvement and openness and discussed project plans. Additionally, an identification of the main challenges and proposed solutions elucidated from each stakeholders’ level (see Table 3). All scaling and reporting were reviewed and agreed by MN and PO in line with the adapted framework, any disagreement was resolved through consensus with the team.

**Table 3:** Project intervention challenges and solutions proposed by stakeholders in rural Cameroon.

Stakeholder (level)	Questions and contextual challenges raised by stakeholders	Project scope and proposed strategies	Observations and important considerations from stakeholders and research team
Secondary recipients	How will health facilities continually navigate poor electricity and internet supply since we have to collect this data electronically?	Project scope did not provide solar system to support facility because this follow-up pilot phase was to understand contextual challenges as we included both French and English districts, pain areas and generate baseline data to support implementation of the digital health project at scale.	Central level stakeholders proposed that many similar projects have come up at the ministry of public health but did not materialize because of some of the issues raised here. Therefore, efforts should be made to ensure this project is followed up to a logical conclusion and scaled up. The BornFyne team should work closely with the directorates involved and the National Information System.
	Some health facilities do not have computers	The project supports health facilities with computers	
	How does the project fit into the existing system of health care in Cameroon, such as the DHIS2	Project integrates elements of the recent WHO DAK for antenatal care which has been harmonized to facilitate interoperability. Interoperability layers will be discussed and developed in the next phase of the project (conditional upon additional funding) to enable a push of data from BornFyne-PNMS to DHIS2	There is a need to discuss with the stakeholders to define specific variables and interoperability aspects
	RMNCH data is usually collected on paper in a form of a physical register. Since BornFyne intends to broaden electronic data collection, are all the variables collected on the physical register included in the BornFyne mobile application?	Most of the variables collected in the registers are included in BornFyne version 1.0. However, given that this is a pilot, and the complexity of introducing such digital transformation intervention, we are using a phased approach and thus we have limited the scope to test usability and fidelity before we expand on all the variables. Currently, the platform collects mostly data for antenatal care collected in the register and more that is not collected in the registers, and it is remodelled to align with the DAK elements	This is a very important solution that has been proposed and the digital platform is still a work in progress as it incorporates the DAK elements which reflects the standard guidelines, this will continuously be updated in consultation with the directorate of family health and the central level stakeholders to capture all the said variables and adapted within the context
Primary recipients	Lack of follow up on client during farming season, the reminder messages will help but not all women owned smart phones	Follow up is ensured by the recruitment of community health workers from localities who are familiar with the women and farming seasons	This is an important equity concern because we understand some households do not own smart phones. This phase of the project doesn’t provide smart phones but will help document this gap and generate data on the willingness to use the digital platform by women. This data will inform the next phase of the project and demand creaiton
	Women and households who do not own mobile phones will be left out of the project	This is an important equity issue that the project took into consideration from the proof of concept and provided smart phones to test the platform in an idle situation. However, in this phase, our goal is to generate data to inform coverage and willingness to use the platform. This information will guide implementation at scale and how to support digital intervention for sustainability	Digital health platforms that use smart phones have important limitations on coverage and use by poor rural women and the need to support poor households with smart phones and or develop strategies to utilize regular phones. Despite high mobile phone penetration, some households and women do not own smart phones.
			Given the complexity of some technology to support services with regular phones, provision of mobile phones to some households is an important component. Continuous engagement with telecommunication companies to facilitate the use of other platforms to address equity concerns is important
	Our community needs a lot of sensitization and education. A lot of women are dying because of ignorant, it is not only about the cost, but there is a general lack of information and follow up	The team has designed community engagement and sensitization plan and will discuss community sensitization strategies with the community health workers for each district and design an approach to educate and sensitize the community based on issues raised during focus group discussions	Massive community sensitization is required in the implementation process of digital health platforms especially at households and social gathering for rural communities and the use of other media coverages
	Concern with network connectivity especially in hard-to-reach areas; and the issue of available airtime on the phones	The research team shared that they were in talks with select network providers and it was the same providers suggested by the community members.	Health facilities do not have internet supply, and this is an important observation and contextual reality. Therefore, digital intervention requires minimal provisions of internet availability to participating health facilities and to the health care providers engaged in the intervention
		Airtime will be provided to health facilities and health providers to support communication during emergencies	
	Concern with range of reproductive age of women to enhance inclusion or otherwise	Project adapted community's evidence of “reproductive age” which was slightly different from WHO recommendation due to escalating pre teenage pregnancy in some of the districts	There are important contextual differences especially in the French and English districts and high rates of pre-teen pregnancy is common in the French district
Primary providers	Sustainability of the project. Will the team leave after this phase of the project, we do not want to start such an exciting project and we are unable to continue because this is something that is common in this system	This is very important, and this is essential and one of the reasons we are engaging everyone on the design and implementing because this project is for your community and to facilitate your daily activity in delivering care.	One of the strategies was the creation of the stakeholder meeting at the central level for continuous update and discussions and feedback. The engagement and partnership with the department of directorate of family health to ensure smooth implementation and feedback
	Problem with low ANC utilization [especially among women “old mothers” (i.e., women who have birthed children before)]	Increasing home visits and educating women on the importance of seeking antenatal care, the use of reminder text messages for follow up and the personalized communication with their health providers	There is need for massive sensitization in the community because women do not attend antenatal care not only due to cost but also cultural reasons and ignorance
	Some of us have not used digital platform to support care, how are we going to adapt?	We have organized a series of training which will involve on one- one and group and peer to peer training to ensure we continuously train and build capacity. We understand it takes time to train and build capacity in using technology platform	The team has established a training module and program to support health providers which will be in both face to-face and audio including training manuals. In addition to series of workshops to support health providers and build sustained capacity of local leaders in using digital platforms
Secondary providers	Problem with selected women who prefer to visit only specific facilities in their catchment areas	Women have a choice to uptake ANC in one facility and deliver in another even when cost of delivery isn’t covered by the project.	Women shop around for ANC and delivery in this context however, with the perception of connecting to their health facility and provider, women saw the need to maintain a particular health facility to ensure they get all the benefit from the BornFyne features as they would not be connected if they change health facility that PNMS is not yet installed in.
		Women were however, encouraged through community health workers to visit intervention-participating facilities	

### Trustworthiness

As is the goal of participatory research, this study looks to attain depth and breadth in understanding of a multifaceted perspective of various study participants’ experiences and perceptions regarding the research topic (26, 27). To account for participants’ prior knowledge, biases, and personal preconceptions of the RMNCH digital health intervention implementation that may be superimposed onto the study, one of the study researchers (MN) bracketed (28) before conducting the stakeholder meetings that gave rise to the discussions. This enabled reflexivity by the interviewers, on their influence on interview participants, their perceptions, and the influence of researchers on their responses regarding the collected data. Researcher triangulation and persistent observation were employed by PO, MN, AWF, VN, FW, OK, and SY to ensure credibility and dependability by reading, re-reading, and cross-checking the data to ascertain correct interpretation of participants’ original views and evaluation of findings as supported by the data received from the participants of the study (29). Constant comparisons and theoretical saturation helped to ensure that the data was methodically representative and explicable (fit and work) (22). The study findings meet the conditions of a quality iterative process—coding and memoing, constant comparisons and theoretical saturation.

## Results

A total of 5 stakeholder meetings were conducted including, one from each district, one central level stakeholder, and a total of 86 stakeholders were identified. Participants in this study all resided in the districts of focus and adequately participated through stakeholder meetings, sufficiently enriching the intervention with their valued perceptions of the BornFyne project.

### Characteristics of identified stakeholders

The study involved several stakeholders identified and categorized based on common interests toward the intervention. All district stakeholder meetings included government officials, the mayor, the divisional officers, religious leaders or representatives, chiefs, quarter heads, community health workers, pregnant women, and health care providers (Table 2).

As indicated above, we used a framework that highlighted the key areas of our study but the six (6) new themes that emerged outside of this framework closely align with the key issues that our paper sought to address. Therefore, the discussion of findings will center on the six (6) major themes that emerged from this research and the key findings from the framework (Tables 1, 3) will be presented in a blended manner as adapted from Namazzi et al. (24). The 6 major themes are namely: (1) situating the agenda; (2) support for the intervention; (3) implementation challenges and solutions highlighted by stakeholders; (4) male involvement; (5) sensitization; and (6) equity concerns.

### Situating the agenda

The project team opened the meeting floor with introductions by name and contributions to the project. Other meeting attendants/participants followed suit and the purpose and importance of the meeting was reached, which was to actively participate and collaborate on this project with the goal to improve access, quality of care and curb maternal and child mortality in the region(s). There was a graphical representation of the roadmap of the project clearly displayed on the board at the meeting venue showing details about the intervention about to kick off. At the central level, a demo version from the proof of concept (version 1.0) was also demonstrated to stakeholders to give them a picture of the platform. All were encouraged to share their thoughts and opinions about the project and the importance of the meeting, which strongly revolved around community participation and continual engagement for improvement. The quotes below highlight their views.

“*I think we will need to keep discussing back and forth and see what is the best. We will keep giving you [project team] feedback. I would like to thank the project team for taking time to explain this exciting project to us. We have also learned a lot in the process, and I think you also did learn from us as well. So, the ball is gradually coming into our court for us to show what we can do and not to disappoint our people*” [DMO, district level].

A second stakeholder further went to highlight the importance of the meeting, thus:

“*I would like to congratulate your team because the message which you have brought to us is very important as it concerns our women and children*” [Church leader, district level].

### Support for the intervention

Generally, there was a strong agreement and support for the project intervention at all levels. However, there were varying positions of influence that emanated from the stakeholders’ engagement and expressed through their body language and verbal communication concerning the project intervention, during the meeting/interviews. Secondary beneficiaries such as church and community leaders were in support of the intervention as they were in great hope for a decline in maternal mortality in the region. Pregnant women and mothers were also found to be highly in support of the intervention as well (see Table 2). As a result of this, one of the outcomes of the central stakeholder meeting was the creation of a WhatsApp group for the BornFyne central level stakeholders which included all the participants that were invited for the stakeholder meeting.

“*I want to thank everyone for coming and I would also like to thank those who wrote this project for choosing our health district because we are sure that at the end it will help to curb down maternal mortality or to put in place strategies that will help us fight maternal and neonatal maternity*” [Male participant].

“*BornFyne PNMS is like a crop that has been brought introduced for us to nurse. The nursery is the Ministry of Public health, and it is now left for the ministry to water the crop until it is ready to be transplanted nationwide*” [Central level stakeholder].

At the central level, the support was seen from diversified perspective, including the need to improve on data and capacity building, strengthening health management information system, and a focus on maternal health to address maternal mortality. Importantly, at the central level, most stakeholders were interested in understanding how the project would facilitate their objective of achieving universal health coverage.

“*This sounds like manna from heaven at a time when the country has just launched the digital framework and looking forward to universal health coverage, an intervention like this which connects to households and the community is something we truly appreciate and helps to support clinical data and monitoring*” [Central level stakeholder]

“*First, I must commend the team for thinking about such a project most importantly that connects the health providers with the community, this is important for our rural women during delivery*” [Central level stakeholder]

### Implementation challenges and solutions highlighted by stakeholders

Issues emanating from the engagement with stakeholders revolved around challenges and proposed solutions that could steer the intervention's direction and inform the intervention. Table 3 below shows a summary of these expressed concerns that arose in the meetings, which varied across stakeholder levels. Concerns ranged from navigating poor electricity and internet supply, to concerns related to the potential increase in workload due to the recording of additional data through the BornFyne-PNMS platform (besides those recorded in the physical registers) to enhance the project's rationale of generating RMNCH data across the country.

### Male involvement

Husbands/male partners to the women involved in the project, expressed their thoughts on wanting to be involved in the project and any other that involves their women. Some women also agreed that if they (men) be involved, it would increase transparency, male encouragement and minimize any unforeseen bottlenecks with the project intervention.

“*You cannot talk to the women alone. When you are talking in the presence of their husbands, the message will go to the man directly, as such the problem will be solved, and he will not one day cause trouble*” [Female participant].

“*That means you will have to encourage your wife that if she finds herself in any situation she can go to a particular facility since we will be providing them transport fare to the said facility*” [Community health worker].

Husbands/male partners, and other men from the community in attendance though in high support, were particular about their involvement in the intervention, expressing that they should always be involved in any project that is targeted at their wives, female partners and/or women in the community.

### Sensitization

The issue of sensitization arose as the solution to issues that bordered on cultural and health-seeking norms and practices. An example was about women who were Muslims in a predominantly Christian community with a renowned practice of late ANC registration, thereby leading to increased maternal mortality. Suggested solution for such concern revolved around sensitization exercises by community health workers of the same faith and also into hard-to-reach areas which looked to enhance access for women. Similarly, when women expressed concerns about knowledge and access to family planning after putting to bed, sensitization was proffered as a possible solution to enlighten them available resources in that regard.

“*Generally, it is believed that family is most important to pregnant women because there are some women who get pregnant and before they menstruate [postpartum], they have another unplanned pregnancy as such during pregnancy from the family planning education, they will get information about different options and that after birth, exclusive breastfeeding will help them not to get pregnant again or they can decide which other method to take to avoid an unwanted pregnancy. That is the reason why although the woman is pregnant, it is very important for her to know about the family planning*” [Male participant].

### Equity concerns

The stakeholders were interested in understanding how the project will benefit women who do not own smart phones and their households. This was very interesting and was relevant to the project team (see Table 3). Discussions on the importance of generating data that will inform the need for supporting digital projects with smart phones arose. In the same vein, participants expressed their concerns on the limited number of households without access to smart phones and the need to consider this disparity. Participants equally presented differences in access to antenatal care by households due to unawareness of antenatal services, distance and cultural attributes (see Table 3).

Important differences were observed and noted during the stakeholder meetings in the French and English districts versus rural and urban settings. In the French districts, participants reported high rates of pre-teen pregnancies in their region and the need to sensitize the entire community in relation to the use of mobile phones for the project. The following remark was raised by stakeholders from the French district.

“*If you are providing mobile phones to the women in this district, all the young girls in this village will get pregnant to get this mobile phone.*”

Another difference was the late registration of antenatal care reported in the French districts. These issues are further explored in focus group discussions in subsequent studies related to the project. In the English districts, the aspect of “shopping around” by women during antenatal care was reported as women would prefer to by-pass health facilities to go further away from their homes for their antenatal care.

## Discussion

This study charts a direction that is critical in digital health delivery of RMNCH in a rural and low-income community and describes the important iterative stakeholder input throughout the study. In line with a study that showed that the involvement of diverse stakeholders enriched the implementation of technology for an aging population (30), our engagement of a diverse range of stakeholders showed the same. From the analytical grid that summarized stakeholder's level of influence and acceptability of the BornFyne-PNMS intervention, most of the stakeholders interviewed were supporters of the intervention because of its benefits to the community and country at large. The level of agreement of stakeholders’ acceptability of the intervention considered was high; no stakeholders were thought to disapprove. In terms of influencing the project, our study points to the high support and high influence from local community leaders and chiefs. Such high influence and support were effective in contributing to community participation in the program, which was similar to the findings of a maternal and child health stakeholder study in Uganda (24). In the same analytic grid, we see a high influence among district health teams across the regions, by way of their upward influence on policy formulation in the districts. The DMOs and health facility directors played a key role in facilitating the integration of the intervention in the existing health system. The health facility directors are able to ensure that electronic RMNCH data are collected through the BornFyne-PNMS digital platform and reported alongside those collected on paper. This way, it was recognizable by all across the districts that electronic data was valid and recognized on a systems level. This tied into the WHO's Service Availability and Readiness Assessment to the Harmonized Health Facility Assessment as a key product of the health data collaborative across Cameroon (31).

Namazzi and colleagues reported on the process and results of their stakeholder analysis for their MNH project with the objective to assess and map out stakeholders’ interests, influence/power, and position in relation to their community based MNH interventions; as well as utilizing stakeholders’ input to inform the refinement and implementation strategy of their intervention. Similarly, our paper describes important lessons on the levels of stakeholders’ direct or indirect influence of the BornFyne-PNMS intervention in the area of RMNCH digital intervention, as well as to elucidate ideas from stakeholders to inform the implementation of our BornFyne PNMS intervention. This stakeholder analysis outlines lessons that are useful for informing RMNCH digital health program interventions in similar LMIC community settings. The involvement of a diverse range of stakeholders (including local stakeholders) throughout each stage of the project, promoted acceptability, ownership, usable knowledge, and community participation. This was similar to findings from Obegu and colleagues on the usable knowledge and enhanced participation that is generated from involving relevant stakeholders in community-based research/programs (16). Stakeholder involvement from the onset of the project is important in the design, implementation and uptake of digital health intervention.

The emergent challenges and solutions of implementation from the diverse stakeholders will evidently enrich the implementation process. In particular, the intervention was initially designed to capture real life RMNCH data but without including all the variables that are found on the paper register (existing system of RMNCH data collection in Cameroon). Meeting with stakeholders informed the addition of some of these variables, which will further enhance the RMNCH electronic data collection in line with health information systems strengthening in Cameroon. Furthermore, proposed solutions to raised potential challenges of implementation that were elucidated to inform the project at scale and sustainability of the project. The call for continuous stakeholder engagement gave rise to continuous stakeholder meetings throughout the implementation phase of the project intervention. During these meetings, we learned about the importance of sensitization as an acceptable solution to issues that bordered on religio-cultural health-seeking norms and practices. For example, a population of Muslim women in the community who also lived in the hard-to-reach areas where ANC services were non-existent, were reached out to by community health workers of the same faith and educated on the availability and access of the mHealth intervention. A finding from this study that looks to inform similar mHealth interventions in LMICs with similar cultural settings as rural Cameroon, is that men are perceived as head of the homes and by norms given a central place in society. This means that husbands/male partners and men in general are ‘gatekeepers’ whose involvement will ensure a smooth implementation of digital intervention targeted at women and children in community settings. This finding was in tandem with a study that saw an increased promotion of maternal and child health when men were involved (32). This aspect of male involvement was further explored in the project using focus group discussions and will be discussed in subsequent papers. In addition, the late registration of ANC in the French districts and the need for community sensitization were identified as important aspects that informed strategic intervention approaches in the implementation phase. The attitude of shopping around for antenatal care was reported in the English district. This shopping around during ANC corroborates with previous study from Cameroon which was related to the attitude of health workers not respecting privacy and confidentiality of patient record and cost (33). This aspect was further explored in detail during focus group discussions in subsequent papers.

## Strengths and limitations of the study

The participatory research component of this study is considered a strength of this study as evidence generated from this project and by extension this study, will inform government strategies and policies set out in the national digital health framework, highlighting community perspectives in the ongoing digital health intervention. Furthermore, by way of the participatory research, using information gathered during stakeholder meetings to inform intervention strategies through the digital platform ensured that the platform was adapted to suit the local needs of community members. Most LMIC communities in sub-Saharan Africa (SSA) tend to have contextual similarities (including resources, norms, values, and practices). As such, the findings of this study are easily transferable to other similar contexts in SSA. It is important to note that the study was not designed to assess differences across sociodemographic characteristics of the stakeholders (e.g., policymakers and health workers, French and English districts), hence the lack of demographic information of stakeholders. The sampling for this study relied solely on letters of invitation sent to identified stakeholders and word-of-mouth invitations. This may constitute a limitation in that, some potential participants may have been left out by way of the above sampling methods.

## Conclusion

This paper and its adapted framework will help LMICs implement and evaluate the digital data collection tools in the healthcare delivery system, as WHO is also demanding for digital data with the launch of the WHO digital adaptation kit for antenatal care. When LMIC data of this nature is available in digital format, it will be readily available for review and analysis, allowing for statistical projections of mortality and morbidity of communities where applicable. This will in turn strengthen the health system in modifying programs targeted for morbidity and mortality reduction. Ultimately, the strategy of stakeholders’ involvement in the BornFyne-PNMS implementation charts a direction for ownership and sustainability in the strengthening of Cameroon's health information system.

## Data Availability

The original contributions presented in the study are included in the article/supplementary material, further inquiries can be directed to the corresponding author.
